# Imaging of Activated T Cells

**DOI:** 10.2967/jnumed.122.264097

**Published:** 2023-01

**Authors:** Mohammad O. Sako, Benjamin M. Larimer

**Affiliations:** 1 Department of Radiology, University of Alabama at Birmingham, Birmingham, Alabama;; 2 O’Neal Comprehensive Cancer Center, University of Alabama at Birmingham, Birmingham, Alabama; and; 3 Graduate Biomedical Science Program, University of Alabama at Birmingham, Birmingham, Alabama

**Keywords:** PET, T cells, immunotherapy, cancer

## Abstract

The adaptive immune response plays a critical role in detecting, eliminating, and creating a memory toward foreign pathogens and malignant cells. Demonstration of the specific and effective target killing of T cells in cancer has reignited interest in the study and therapeutic manipulation of the interaction between tumor and immune system. To both improve therapeutic efficacy and reduce adverse events, accurate monitoring of the activation of T cells is required. Several approaches to monitoring not just the presence, but importantly the activation, of T cells have been developed. Here, we review the recent advances in T-cell activation imaging and future directions for potential implementation into clinical utility.

T cells represent the target of many immunotherapies, including checkpoint inhibitors, bispecific T-cell engagers, and immune agonists (1). Additionally, T cells themselves have been investigated as therapies using either chimeric antigen receptor addition or autologous adoptive transfer in hematologic malignancies (2). In solid tumors, although some success has been achieved by checkpoint inhibitors targeting programmed death protein 1 (PD-1), cytotoxic lymphocyte antigen 4, and lymphocyte activation gene 3, most approaches have not increased overall survival (3*,*^4^). Furthermore, approaches to activate T cells to kill cancer can induce severe immune-related adverse events and, in some cases, lead to death (5).

The dynamic and spatially heterogeneous nature of immune responses makes predicting and monitoring immune activation difficult in vivo. Currently, imaging approaches to monitoring response include quantifying therapeutic targets (i.e., programmed death ligand 1), the presence of immune cells (i.e., CD8), or T-cell activation. Although the presence of a therapeutic target and T cells are necessary, they alone are not sufficient, as other immunosuppressive factors may abrogate antitumor response. T-cell activation ultimately represents the integral of the pro- and antitumoral signaling within a tumor, providing a simplified readout of a complex process, including antigen presentation, T-cell priming, antigen recognition, and appropriate costimulatory signal transduction (Fig. 1) (6). Given the whole-body, noninvasive, and quantitative nature of PET imaging, many groups have developed novel imaging agents for interrogating various aspects of the T-cell response. In this review, we cover the recent advances in imaging of activated T cells. In general, the approaches to monitoring activated T cells have focused on several differentiating aspects of activated T cells, including metabolism, secreted molecules, and surface receptors. The strengths and weaknesses of each target, as well as current imaging agents and approaches, will be reviewed. Finally, future directions and potential next-generation targets will be examined.

**FIGURE 1.**
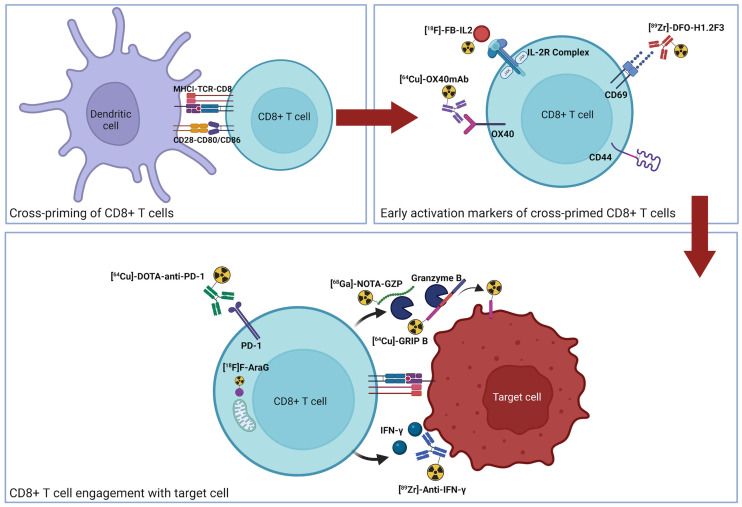
Graphical representation of T-cell activation and corresponding PET imaging agents. Cross-priming of naïve T cells (top left) leads to activation (top right). On recognition of cognate antigen, functional release of granzyme B and IFN-γ is induced (bottom). GRIP = granzyme targeting restricted interaction peptide specific to family member B; mAb = monoclonal antibody; MHCI = major histocompatibility complex I; TCR = T-cell receptor.

## METABOLISM

Arabinosylguanosine (AraG), a nucleoside analog, has been shown to exert selective killing against T lymphoblasts (7). The nucleoside analog undergoes phosphorylation by deoxyguanosine kinase, in particular when there are lower concentrations of the substrate in the mitochondria and the cytoplasm (8). Namavari et al. (9) first synthesized the tracer 2′-deoxy-2′-^18^F-fluoro-9-β-d-arabinofuranosylguanine (^18^F-AraG) and demonstrated uptake in activated T cells; however, Levi et al. (10) were the first to evaluate immune checkpoint blockade (ICB)–related changes in immune response using the tracer. In the study of Levi et al., longitudinal ^18^F-AraG imaging in a murine sarcoma virus-moloney leukemia virus–induced rhabdomyosarcoma model from weeks 1–3 followed the course of immune activation, with a stable increase in tumor and draining lymph node signal over time. ^18^F-FDG tumor signal, however, followed the pattern of tumor growth, starting from an initial increase to a decline from weeks 1–3. Furthermore, ^18^F-AraG PET imaging of MC38-bearing mice showed a significant difference in tumor uptake between baseline and only 2 d after initiation of treatment with anti-PD-1 therapy, with a nonsignificant increase in nonresponders. Overall, the tracer predicted the response to anti-PD-1 therapy early in the course of the disease. In a separate study (11), Levi et al. attempted to characterize crucial anticancer immune cell infiltration in the tumor microenvironment by PET imaging with ^18^F-AraG and changes associated with chemotherapy. Murine syngeneic tumors with varied immune infiltration profiles had differing tracer uptake in both tumors and tumor-draining lymph nodes. A significant positive correlation was reported between tracer uptake and the number of CD8-positive, PD-1–positive cells. Furthermore, the PET signal was significantly higher after the introduction of immunogenic cell death–inducing chemotherapy than at baseline in both MC38 and A9F1 murine tumor models (11). Outside cancer immunotherapy, Levi et al. (12) demonstrated the capability of the tracer to detect sites of early T-cell activation in a tested mouse model of acute graft-versus-host disease. The significant preclinical evidence for this tracer has led to clinical investigation in several ongoing trials focused on non–small cell lung cancer; however, to date only biodistribution and safety in healthy volunteers (NCT02323893) have been published.

## SECRETED MOLECULES

### Granzyme B

Granzyme B, a serine protease that induces apoptosis in target cells, is released by activated CD8-positive T cells and natural killer cells, in addition to some immunosuppressive cells (13). Granzyme B peptide (GZP) was the first probe used to image the serine protease, by Larimer et al. (14). After injection of the radiolabeled tracer ^68^Ga-NOTA-GZP into mice, PET imaging was able to predict response to ICBs before divergence into responders and nonresponders based on tumor volumes, with the former having higher GZP uptake (14). In addition, further studies were performed to evaluate the ability of GZP to predict the response to combination therapy with both ICBs and chemotherapy (15). In an effort to explore cancer immunotherapy–driven adverse events, Ferreira et al. (16) investigated the role of GZP PET imaging in a regulatory T-cell–depleted model of immunotherapy-induced adverse events. The model mice demonstrated significantly increased uptake of the tracer in the colon in comparison to appropriate controls, and this increase was reversed by the addition of steroids, supporting the potential translational capability of GZP to monitor immune-related adverse events.

Granzyme B proteolytic activity has also recently been imaged by Zhao et al. (17) using the multidomain tracer granzyme targeting restricted interaction peptide specific to family member B. On cleavage of a granzyme B–specific domain in the center of a peptide, a radiolabeled ubiquitous cell-binding domain is unmasked and binds irreversibly to the nearby phospholipid bilayer. The granzyme B–specific activation of binding allows measurement of granzyme B activity over prolonged periods on radiolabeling with ^64^Cu. The tracer accumulated in tumors both more quickly and with higher uptake in ICB-treated than vehicle-treated mice. Furthermore, spleen uptake of the tracer was higher with ICB treatment. To show utility in noncancer T-cell activation applications, increased granzyme B activity in the lungs, thymus, and spleen of mice was demonstrated after intratracheal instillation of lipopolysaccharides.

Clinically, PET imaging of granzyme B has had an early clinical readout from Zhou et al. (18) using a ^68^Ga-labeled peptidomimetic. Two patients, one with lung adenocarcinoma and another with sarcomatoid carcinoma of the lung undergoing checkpoint blockade, were imaged after treatment initiation. The lung adenocarcinoma, a partial metabolic responder, had a granzyme B PET tumor SUV_max_ of 4.1 and a tumor-to-blood ratio of 1.2, whereas the sarcomatoid carcinoma was determined to be a partial nonresponder, with a tumor SUV_max_ of 2.0 and a tumor-to-blood ratio of 0.8. These data are preliminary but consistent with preclinical studies demonstrating the predictive capabilities of granzyme B PET imaging.

### Interferon-γ (IFN-γ)

IFN-γ is a cytokine released by a myriad of cells, including cytotoxic T lymphocytes, T-helper 1 cells, natural killer T cells, B cells, and natural killer cells. The downstream effects of the cytokine are pleiotropic and can either promote or inhibit inflammation (19). Gibson et al. (20) first investigated the role of IFN-γ PET imaging in predicting response to anticancer immunotherapy. An anti-IFN-γ monoclonal antibody radiolabeled with ^89^Zr was used in several cancer models. Increased localized tracer uptake in the spleen of mice that received CpG-oligodeoxynucleotide to induce IFN-γ release was observed by PET imaging 72 h after injection of the tracer. In another experiment, the tracer was injected in mice bearing neu-positive TUBO cancer cells to uncover active immune response after administration of 2 doses of human epidermal growth factor receptor 2 (HER2)/neu DNA vaccines. Mice that received the vaccine started to respond after the second dose and had tumors with significantly higher uptake of the tracer than control mice. Furthermore, higher uptake of the tracer after HER2/neu DNA vaccination and subsequent Treg depletion was shown in a spontaneous model of cancer using neu transgenic mice. An inverse relationship between tracer uptake and tumor volume was observed in TUBO tumors implanted in BALB/c mice that received the vaccine. Taking the findings together, the study proposed a role for IFN-γ PET imaging in evaluating active T-cell–mediated anticancer immunity in situ.

### Interleukin-2 (IL-2) Receptor

IL-2 is a cytokine that affects the development and differentiation of several T-cell subsets, such as CD8-positive T cells, natural T-regulatory cells, and CD4-positive T-helper cells, with resultant either pro- or antiinflammatory downstream effects depending on the subset of the target cell. The cytokine, however, is particularly essential for the proliferation, effector function, and memory development of CD8-positive T cells (21). The cytokine binds to its receptor (IL-2R), which is a complex formed by 3 different subunits in its high-affinity form, namely IL-R2α, IL-2Rβ, and IL-2Rγ (22). For PET imaging of IL-2R, IL-2 was radiolabeled with ^18^F by forming the tracer *N*-(4-^18^F-fluorobenzoyl)IL-2 (^18^F-FB-IL2) (23). Later, van der Veen et al. (24) synthesized 2 IL-2–radiolabeled tracers, ^68^Ga-Ga-NODAGA-IL2 and ^18^F-fluoride-(restrained complexing agent)-IL2 (^18^F-AlF-RESCA-IL2), to compare them with the former one and to overcome the hurdles associated with its synthesis. The authors demonstrated that activated human peripheral blood mononuclear cells had higher uptake in vitro of ^18^F-AlF-RESCA-IL2 than of the other IL-2–labeled tracers. A similar trend by the bone marrow and spleen was observed in ex vivo biodistribution experiments. In addition, PET imaging revealed higher uptake of the 3 tracers by subcutaneously inoculated activated human peripheral blood mononuclear cells in severe combined immunodeficient mice than in control counterparts. The study proposed ^18^F-AlF-RESCA-IL2 as a potential candidate for PET imaging of IL-2R–expressing cells.

In a nonrandomized, open-label clinical trial (25), a small sample of patients with stage IV metastatic melanoma was transfused with ^18^F-FB-IL2 before and through treatment with ICBs in either combination therapy or monotherapy to assess changes in immune response by PET imaging (NCT02922283). The SUV_max_ was low for tumors and dropped slightly from 1.8 at baseline to 1.7 at later time points while on therapy for patients who had both scans. No correlation between response and changes in uptake were observed. The SUV_mean_ in the bone marrow and spleen was reported to be 2.5 and 10.9, respectively. However, the authors did not determine the exact cause behind the inability of their imaging study to detect changes in immune response in tumors after treatment.

## SURFACE RECEPTORS

In addition to changes in T-cell metabolism, T cells also undergo changes in the surface expression of specific molecules. Although some of these cell surface proteins indeed function to attenuate activation, their presence nonetheless indicates activation of immune cells. Cell surface receptors, in comparison to secreted molecules, represent an attractive imaging target as they remain tethered to the cell surface and can be expressed in high concentrations, whereas secreted markers may be diffuse and limit maximal signal concentration.

### PD-1

PD-1 is a checkpoint receptor that is upregulated in T cells after T-cell receptor stimulation (26). Inhibition of PD-1 and its cognate ligand programmed death ligand 1 have revolutionized cancer therapy, as reinvigoration of activated T cells by PD-1/programmed death ligand 1 blockade can generate durable remission of metastatic disease (27*,*^28^). PD-1 imaging was first performed in the Gambhir lab using a ^64^Cu-labeled antimouse IgG (29). In this seminal work, Natarajan et al. demonstrated persistent accumulation in the lymphoid organs and tumors of transgenic mice bearing B16-F10 melanoma tumors. Subsequent approaches to PET imaging of PD-1 have included using therapeutic antibodies, including ^89^Zr-df-pembrolizumab and ^89^Zr-nivolumab, in humanized mouse models and ^89^Zr-nivolumab in nonhuman primates (30–32).

Clinical PD-1 PET imaging was first reported by Niemeijer et al. (33) in 2018 using ^89^Zr-nivolumab. Like preclinical studies, high uptake was visualized in the spleen, liver, and tumor over time. Tumors with immunohistochemical confirmation of PD-1 presence had statistically significant increases in the SUV_peak_ signal of ^89^Zr-nivolumab, and subsequent responding tumors also had significantly higher levels of tracer accumulation. In addition to ^89^Zr-nivolumab, ^89^Zr-pembrolizumab has been investigated clinically (34). Eighteen patients, 11 with melanoma and 7 with non–small cell lung cancer, were imaged on days 2, 4, and 7 after injection of 0.37 MBq of ^89^Zr-pembrolizumab plus 2.5 or 7.5 mg of unlabeled pembrolizumab. The results were similar to those for ^89^Zr-nivolumab, with marked uptake in the spleen, lymph nodes, tumor, and liver. Overall, uptake correlated significantly with both progression-free and overall survival. To date, PD-1 represents the most advanced PET imaging marker. Although the pharmacokinetics of antibody-based imaging make routine clinical practice challenging, the results of these studies demonstrate significant potential for PD-1 as a predictive biomarker.

### OX-40 and Inducible T-Cell Costimulator

OX-40 (CD134) is a member of the tumor necrosis factor receptor superfamily and has been reported to be restricted to antigen-specific activated T cells. The first report (35) of OX-40 PET imaging used a ^64^Cu-DOTA–conjugated murine antimouse OX-40 monoclonal antibody. In this study, mice bearing dual A20 tumors were administered an in situ CpG oligonucleotide vaccine and imaged 2 and 9 d after therapy. Significant differences in uptake were quantified between treated and vehicle tumors at 2 d after injection, and unsupervised hierarchic clustering indicated that uptake was predictive of response. A similar approach has been used for glioblastoma; however, interestingly, tumor demonstrated higher accumulation in the vehicle-treated mice than in the vaccinated mice (36). Lymph node and spleen values were higher in the vaccinated mice, suggesting greater involvement of the peripheral lymphatic system in the efficacy of an intramuscular tumor vaccination approach. The differences demonstrate the varied roles and importance of spatial localization in monitoring activated T cells. Outside cancer, OX-40 PET imaging has also been used to diagnosis acute graft-versus-host disease, with significant increases in the lungs and liver of mice transplanted with human peripheral blood mononuclear cells (37). Similar to OX-40, an ^89^Zr-deferoxamine–inducible T-cell costimulator antibody has been developed to track T-cell activation after introduction of a stimulator-of-interferon-gene agonist and checkpoint blockade (38). In a syngeneic lung cancer model, PET imaging revealed rapid and persistent upregulation after stimulator-of-interferon-gene agonist administration that correlated with changes in tumor volume.

### CD69

CD69 is an early cell surface marker of activated T cells. It functions as a signal transducer to promote proliferation but can also be found on monocytes, neutrophils, and eosinophils, in addition to constitutive expression on mature thymocytes and platelets. The first targeted imaging agent for CD69 was an Affibody (Affibody AB), Z_CD69:2_, that bound to both murine and human CD69 (39). The Affibody was conjugated to DOTA and radiolabeled with ^111^In for SPECT imaging. The tracer cleared rapidly from the blood through renal excretion and accumulated in a lymph node of a healthy rat and at the site of allograft rejection in a mouse model. More recently, an ^89^Zr-labeled antibody was used for PET imaging of CD69 (40). The authors examined the ability of the tracer to differentiate responding from nonresponding syngeneic CT26 tumors during checkpoint blockade and demonstrated significant differences in the blood, spleen, and tumor between treated responders and nonresponders. Furthermore, the authors confirmed target specificity using autoradiography and immunohistochemistry, providing a robust correlation between PET signal and tissue protein expression.

## FUTURE DIRECTIONS

To date, a significant amount of preclinical evidence has been generated to support imaging of activated T cells to predict response to checkpoint blockade in tumors or to monitor the development of autoimmune disease. Early clinical trial results have been mixed, with PD-1 PET imaging correlating with response and IL-2 PET imaging demonstrating no correlation. In addition, some agents, including ^18^F-AraG (NCT04726215, NCT05096234) and NOTA-human GZP (NCT04169321), are currently under investigation in clinical trials. One current limitation of several preclinical targets (IFN-γ, OX-40, CD69) is the lack of human-specific probes for translation to clinical trials. The temporal dynamics of the immune system may make antibody imaging challenging because of the extended timelines between injection and image collection. Ideally, high-affinity ligands for these targets with rapid pharmacokinetics would allow for robust implementation into a clinical imaging paradigm.

The opportunity for one or more of the current imaging approaches to impact cancer immunotherapy remains high. Approaches that stratify early response in individual patients or provide quantitative assessments of novel single-agent or combination immunotherapies in early-phase clinical trials would provide the greatest immediate impact on cancer treatment. Continued testing of probes in clinical trials, in addition to translation of promising targets, is necessary to advance the field.

## DISCLOSURE

Benjamin Larimer is a consultant for and shareholder in Cytosite Biopharma Inc. No other potential conflict of interest relevant to this article was reported.
